# Urinary bladder invasion secondary to dissemination of gallbladder cancer: an unusual presentation

**DOI:** 10.1007/s12328-026-02324-1

**Published:** 2026-04-13

**Authors:** Ryotaro Watanabe, Tatsuya Koshitani, Shinsuke Hashidume, Keiu Kasho, Akitaka Yokomura, Hiroshi Takihara, Takahiro Takemoto, Masaaki Itoh, Naoki Nakajima

**Affiliations:** 1 Department of Gastroenterology, Uji Tokushukai Medical Center, 145 Ishibashi Makishima-Cho Uji City, Kyoto, 6110041 Japan; 2 Department of Gastroenterology, Mimihara General Hospital, Sakai, Japan; 3 Department of Urology, Uji Tokushukai Medical Center, Kyoto, Japan; 4 Department of Pathology, Uji Tokushukai Medical Center, Kyoto, Japan

**Keywords:** Gallbladder cancer, Urinary bladder invasion, Peritoneal dissemination, Urachal carcinoma

## Abstract

Gallbladder cancer is an aggressive malignancy that is often diagnosed at an advanced stage, with frequent metastatic spread. Urinary bladder invasion caused by disseminated gallbladder cancer is extremely rare. A 56-year-old man presented with gross hematuria. Imaging revealed tumors in the gallbladder and urinary bladder with peritoneal dissemination. Transurethral resection of the urinary bladder tumor showed adenocarcinoma. Immunohistochemical analysis demonstrated positivity for CK7 and CDX2 and negativity for CK20, findings inconsistent with urachal carcinoma. Biopsy specimens from the gallbladder tumor and bile duct stricture revealed poorly differentiated adenocarcinoma with an identical immunohistochemical profile, leading to a diagnosis of urinary bladder involvement from gallbladder cancer. Despite systemic chemotherapy, the patient died 10 months after treatment initiation. Pathological autopsy confirmed urinary bladder invasion caused by peritoneal dissemination. This case illustrates a rare presentation of gallbladder cancer with urinary bladder invasion causing hematuria and highlights the importance of considering disseminated disease when evaluating urinary bladder tumors in patients with gallbladder cancer.

## Introduction

Gallbladder cancer often progresses without showing any symptoms and is frequently diagnosed at an advanced stage. Furthermore, the gallbladder has a thin muscular layer and partially absent serosa, which makes it prone to distant metastasis. Consequently, the prognosis for patients with gallbladder cancer is extremely poor [1]. The liver and lymphatic system represent the predominant targets of metastasis in gallbladder cancer, while distant metastases often occur in the lungs and bones [2]. However, the urinary bladder invasion caused by dissemination of gallbladder cancer is exceedingly rare. Here, we report a case of gallbladder cancer presenting with hematuria due to urinary bladder invasion, in which pathological autopsy clarified the disseminating spread.

### Case report

A 56-year-old man developed gross hematuria three weeks before presentation and was referred to the urology department of our hospital. Contrast-enhanced CT revealed a urinary bladder tumor accompanied by gallbladder wall thickening. The patient was subsequently referred to our department for further evaluation. He had no significant past medical history or medication history. Physical examination findings were unremarkable. Laboratory findings included mild elevation of biliary enzymes (ALP 597 IU/L, γ-GTP 300 IU/L) and serum CA19-9 (175 U/mL), with a normal total bilirubin level. Urinalysis showed occult blood.

**Fig. 1:**
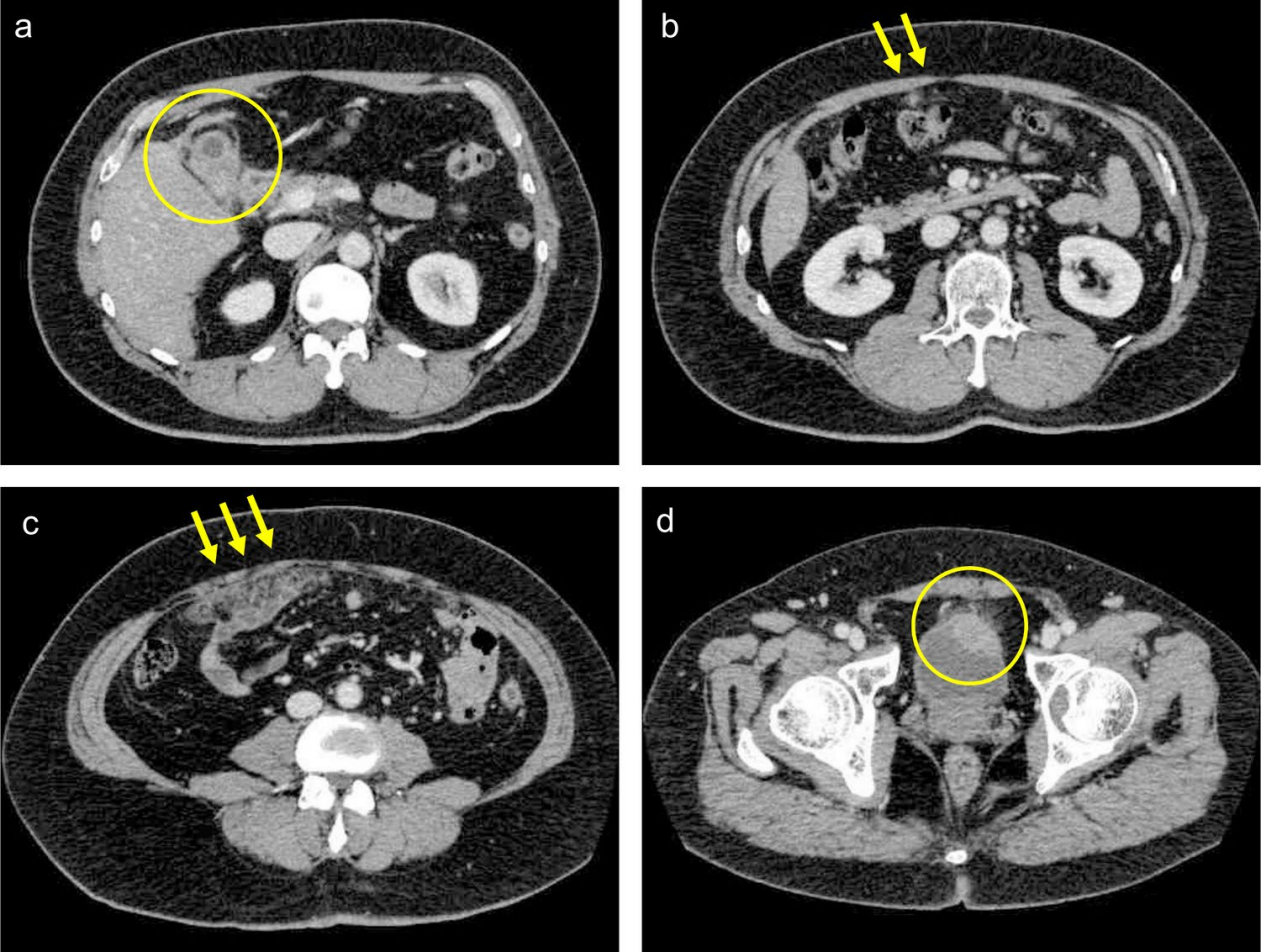
Contrast-enhanced abdominal CT findings. **a** Irregular wall thickening of the gallbladder. **b** Multiple peritoneal nodules. **c** Omental thickening. **d** A urinary bladder tumor

**Fig. 2:**
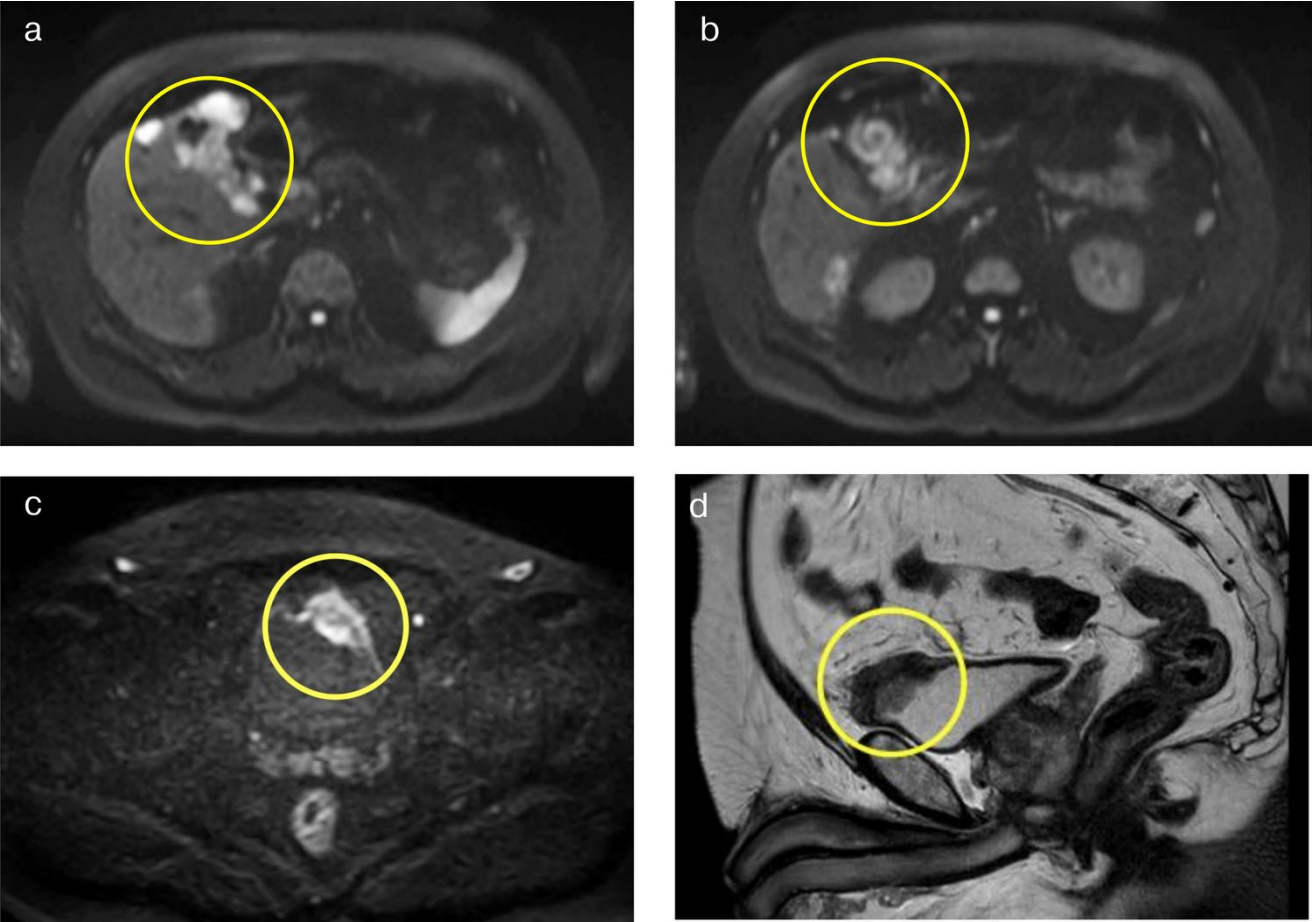
MRI findings. **a ~ c** Diffusion weighted images: **a. b.** Diffusion restriction in the gallbladder wall and adjacent liver. **c** Diffusion restriction in the urinary bladder mass with irregular wall thickening. **d** T2-weighted image. The mass was located at upper ventral side of the urinary bladder in the sagittal view

Contrast-enhanced CT demonstrated irregular thickening of the gallbladder wall with adjacent hepatic low attenuation, peritoneal nodules, omental thickening, and a ventral urinary bladder mass (Fig. 1). MRI revealed diffusion restriction in the gallbladder wall, adjacent liver and urinary bladder wall, with loss of signal at the cystic duct confluence on MR cholangiopancreatography which suggested bile duct invasion by gallbladder tumor (Fig. 2).

**Fig. 3:**
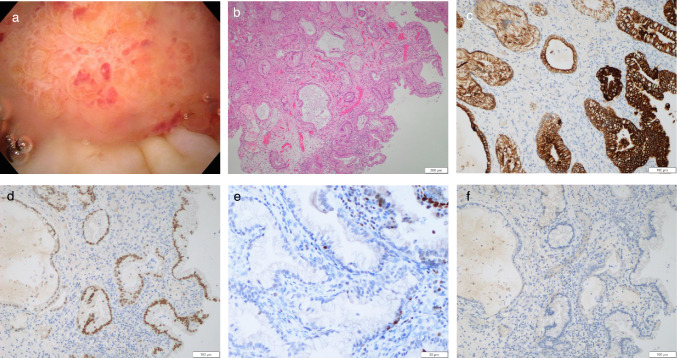
TUR-BT findings. **a** Cystoscopy revealed a protuberant mass on the upper bladder wall. **b** Microscopic examination showed atypical glandular epithelial cells forming variably sized tubular structures within the stroma, consistent with well-differentiated adenocarcinoma. **c ~ f** Immunohistochemical staining: **c.** CK7 positivity, **d.** CDX2 positivity, **e.** GATA3 negativity, **f.** CK20 negativity

For the urinary bladder tumor, transurethral resection of the bladder tumor (TUR-BT) was performed for histopathological diagnosis and staging. Cystoscopy revealed a protuberant mass on the upper bladder wall. Histopathological examination showed a well-differentiated adenocarcinoma composed of atypical glandular cells forming tubular structures with invasion into the muscularis propria. Immunohistochemical staining revealed CK7 positivity, CDX2 positivity, GATA3 negativity and CK20 negativity, which was atypical for primary bladder carcinoma or urachal carcinoma (Fig. 3).

**Fig. 4:**
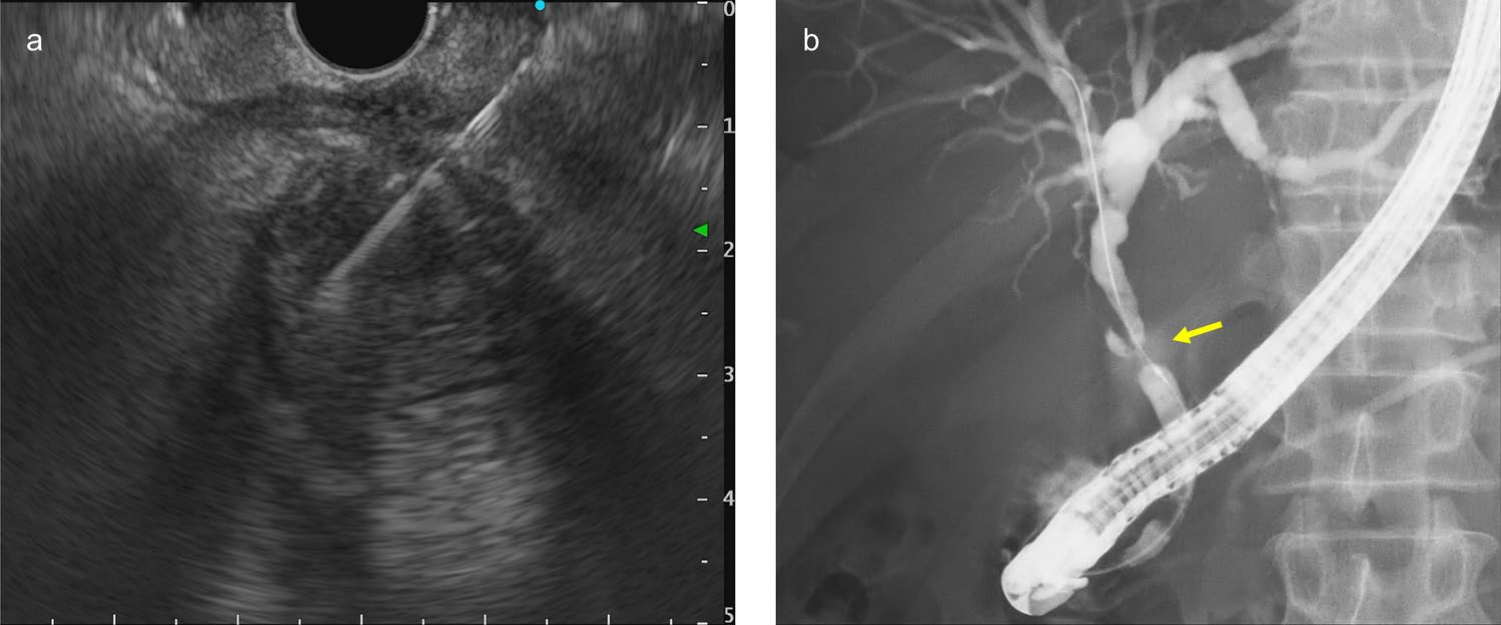
**a** EUS-FNB was performed for the gallbladder wall thickening. **b** ERC showed a bile duct stricture at the cystic duct confluence. Biopsies were taken

**Fig. 5:**
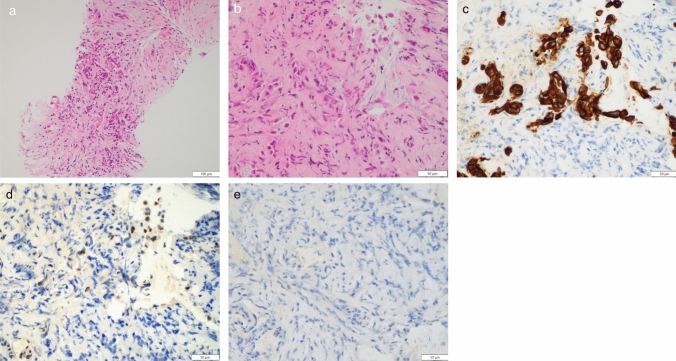
**a** EUS-FNB specimen. **b** Bile duct biopsy. Microscopic examination showed scattered atypical epithelial cells with nuclear atypia infiltrating the stroma, consistent with poorly differentiated adenocarcinoma. **c ~ e** Immunohistochemical staining: **c.** CK7 positivity, **d.** CDX2 positivity, **e.** CK20 negativity

EUS-guided fine needle biopsy (EUS-FNB) was performed for the gallbladder wall thickening using a 22-gauge needle and ERCP-guided forceps biopsy was done for the bile duct stricture (Fig. 4). Both specimens showed scattered atypical epithelial cells with nuclear atypia infiltrating the stroma. Atypical cells formed small nests composed of a few cells and did not form well-formed glands, consistent with poorly differentiated adenocarcinoma. Immunohistochemical staining again revealed CK7 positivity, CDX2 positivity, and CK20 negativity, identical to the urinary bladder tumor (Fig. 5). Based on pathological findings and immunohistochemical concordance, the urinary bladder tumor was diagnosed as secondary involvement from gallbladder cancer. PET-CT revealed extensive hepatobiliary invasion with nodal and peritoneal metastases. The final diagnosis was advanced gallbladder cancer, cT4N2M1, Stage IVB.

After endoscopic biliary stent placement, combination chemotherapy with gemcitabine, cisplatin, and pembrolizumab (GC-PEM) was initiated. Following nine courses of GC-PEM therapy, CT demonstrated progressive disease. Comprehensive genomic profiling revealed no actionable mutations, and the treatment was switched to oral S-1 monotherapy. During the first course of oral S-1 therapy, the patient’s condition rapidly deteriorated, and he died 10 months after the initiation of treatment.

**Fig. 6:**
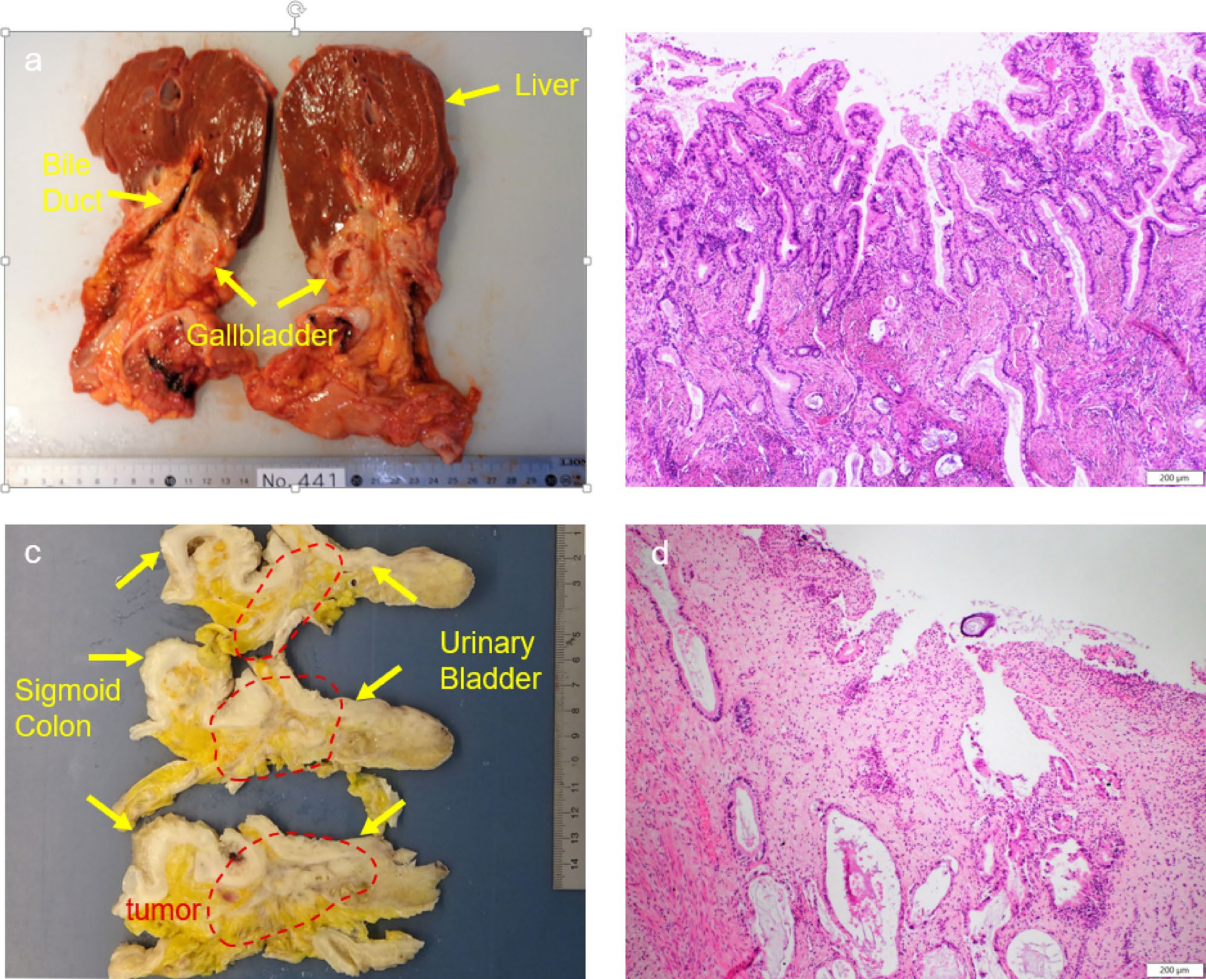
**a** Cross-section of the biliary tree and liver shows a tumor thickening the gallbladder wall. **b** Microscopic examination of the gallbladder wall shows well-differentiated adenocarcinoma with in situ element. **c** Cross-sectional view of the pelvic mass shows a tumor infiltrating the sigmoid colon and urinary bladder. **d** Microscopic examination of the urinary bladder shows well-differentiated adenocarcinoma that is identical to the gallbladder tumor

A pathological autopsy revealed a tumor present in the bladder-rectal fossa, infiltrating the urinary bladder, rectum and sigmoid colon. Histopathological evaluation of the tumor located in the bladder-rectal fossa revealed that the tumor was a well-differentiated tubular adenocarcinoma composed of well-formed glands and small cell nests, and the well-differentiated component invaded the urinary bladder. And histological evaluation of the gallbladder tumor showed that the tumor was a well-differentiated tubular adenocarcinoma composed of well-formed glands and small cell nests that is identical to the tumor of the bladder-rectal fossa (Fig. 6). Based on these findings, the urinary bladder involvement was determined to be caused by peritoneal dissemination from gallbladder cancer.

## Discussion

In an autopsy series of patients with gallbladder cancer, metastases most frequently involved the liver (66%), followed by the lungs (15%), bone system (9%), heart (6%), pancreas (5%), kidneys and adrenal glands (each 5%), as well as the ovary (4%), colon (3%), and brain (2%) [2]. In contrast, the urinary bladder involvement from gallbladder cancer is exceedingly rare, with only one previously reported case in the literature [3].

This patient presented with hematuria, and further evaluation revealed tumors in both the urinary bladder and gallbladder. The differential diagnosis included synchronous primary cancers of the urinary bladder and gallbladder or urinary bladder involvement from gallbladder cancer. Adenocarcinoma of the urinary bladder is rare, accounting for 0.5–2% of all malignant bladder tumors, and is generally classified into urachal carcinoma, primary bladder adenocarcinoma, and metastatic bladder adenocarcinoma based on clinicopathological features. Histologically, these entities are largely indistinguishable [4]. Metastatic disease must be excluded before diagnosing urachal or primary bladder adenocarcinoma [5]. Urachal carcinoma typically shows CK20 positivity [6]. In this case, the urinary bladder tumor showed atypical features for urachal carcinoma, and biopsy specimens from the gallbladder and bile duct demonstrated similar histological and immunohistochemical profiles to those of the urinary bladder tumor. Based on these findings, the urinary bladder lesion was diagnosed as involvement from gallbladder cancer.

The gallbladder tumor was diagnosed as poorly differentiated adenocarcinoma based on EUS-FNB and bile duct biopsy; however, autopsy demonstrated predominantly well-differentiated tubular adenocarcinoma, which was histologically identical to the urinary bladder tumor. This discrepancy may reflect sampling differences between limited biopsy specimens and the whole-tumor evaluation at autopsy, suggesting intratumoral histological heterogeneity.

The urinary bladder can be involved by secondary tumors through direct extension or metastasis from other organs. Among secondary bladder tumors, the primary sites most commonly metastasizing to the bladder are the stomach (4.3%), skin (3.9%), lungs (2.8%), and breast (2.5%) [7]. In a review of urinary bladder metastasis from gastric cancer, the diagnosis of bladder metastasis was metachronous in most cases with an average time of presentation in four years after the primary diagnosis of gastric cancer. The predominant symptom was hematuria. Most patients presented other metastatic sites except for bladder involvement. The most frequent site of metastasis was peritoneum. Macroscopic features of bladder metastasis included diffuse wall thickening in 57% of the cases [8]. Cancer cells can metastasize to the urinary bladder via hematogenous or lymphatic routes, and the urinary bladder can also be invaded directly by peritoneal dissemination. Peritoneal dissemination is considered the most common cause of bladder involvement, often affecting the upper part of the bladder. Diffuse bladder wall involvement can occur through both hematogenous and lymphatic metastasis [9].

Cases of urinary bladder involvement from pancreatic cancer and ampullary cancer have been reported [10]. However, to the best of our knowledge, an autopsy-confirmed case of the urinary bladder invasion caused by disseminated gallbladder cancer has not been documented. In the previous report [3], a urinary bladder tumor was incidentally detected on CT scan during adjuvant chemotherapy following gallbladder cancer surgery. There was peritoneal dissemination, and the urinary bladder tumor was identified in the upper part of the bladder. The inner surface of urinary bladder tumor was covered with normal urothelium, and no tumor exposure into the bladder lumen was observed. In our case, the urinary bladder tumor was exposed in part into the bladder lumen, which likely caused hematuria.

Although the mechanism of urinary bladder involvement from gallbladder cancer remains unclear, the previous report has suggested transperitoneal spread to the urinary bladder serosa with subsequent invasion of the bladder wall. In the present case, autopsy findings clearly demonstrated extensive peritoneal dissemination, with continuous tumor invasion to the urinary bladder supporting peritoneal seeding as the most plausible route of urinary bladder involvement.

We encountered a rare case of gallbladder cancer with urinary bladder invasion presenting as hematuria. Although extremely rare, this case underscores the importance of considering disseminated disease in the differential diagnosis of urinary bladder tumors in patients with advanced gallbladder cancer.
